# Serum and alveolar IGA and IGG2 levels may help to predict ventilator-associated pneumonia outcome

**DOI:** 10.1186/2197-425X-3-S1-A873

**Published:** 2015-10-01

**Authors:** M Gordon Sahuquillo, C Lopez Ferraz, J Bermejo-Martin, M Moreno, I Paredes, E Villarreal Tello, C Calabuig, J Ruiz Ramos, A Cortes, P Geffner, A Castellanos Ortega, P Ramirez Galleymore

**Affiliations:** Critical Care Medicine, Hospital Universitari i Politècnic La Fe, Valencia, Spain; Hospital Clínico Universitario de Valladolid, Unidad de Investigación Biomédica del Clínico, Valencia, Spain

## Introduction

There is an emerging evidence that critical illnes induces an alteration in the immune response of patients that makes them more susceptible to the development of nosocomial infections and influences its clinical response.

## Objectives

The aim of this study is to characterize the immunocompetence status of critically ill patients undergoing mechanical ventilation and to determine impaired immune patterns associated with an increased risk of developing ventilator-associated pneumonia (VAP) and a poorer clinical evolution.

## Methods

All immunocompetent patients over 18 years old admitted to the Intensive Care Unit of the Hospital Universitario and Politecnico la Fe (Valencia, Spain), undergoing mechanical ventilation for more than 48 hours were included. Patients with clinical suspicion of ventilator-associated respiratory infection were considered as cases and compared with a selection of controls matched by age, comorbidities, severity scales and days of mechanical ventilation. At the moment of intubation and each 72 hours until the moment of extubation, clinical surveillance was performed and respiratory samples (mini-bronchoalveolar lavage, mini-BAL) were taken to evaluate clinical and microbiological response. Samples of plasma and mini-BAL were also taken for immunoglobulins IgG1 - IgG4, IgM, IgA and IgE determination. Comparison of numerical and categorical variables was performed with the Mann-Whitney U test. Results were expressed as medians with interquartile (25% to 75%) ranges (IR) in brackets.

## Results

16 cases and 14 controls were considered for analysis. Response to antibiotic treatment was evaluated in cases and patients were classified in responders (7 cases) and non-responders (8 cases). Initial empirical antibiotic treatment was more accurate in responders patients (85.7% vs. 50%; p 0.559). At the moment of orotracheal intubation and beginning of mechanical ventilation, VAP patients showed lower serum IgA levels (284 mg/dl [162 - 664] vs. 663 mg/dl [475 - 1,259]; p 0.049) (figure A), lower serum IgG1 and IgM and lower respiratory levels of IgG1, IgG2 and IgA, without statistical signification. At the moment of VAP diagnosis, responders patients showed higher levels of IgG2 (123 mg/dl [64 - 235] vs. 41 mg/dl [32 - 60]; p 0.037) (figure B), lower serum IgG1 and IgM (without statistical signification) and higher levels of IgA (1,900 mg/dl [536 - 1,900] vs. 22 ng/ml [3 - 589]; p 0.036) (figure C).

## Conclusion

Our findings show the protective role of IgA and IgG2 against the inflammatory aggression of mechanical ventilation and in the response against ventilator-associated pneumonia.

## Grant Acknowledgment

This study was supported by the Spanish Society of Pneumology and Thoracic Surgery (SEPAR 129/2011).

**Figure 1:**
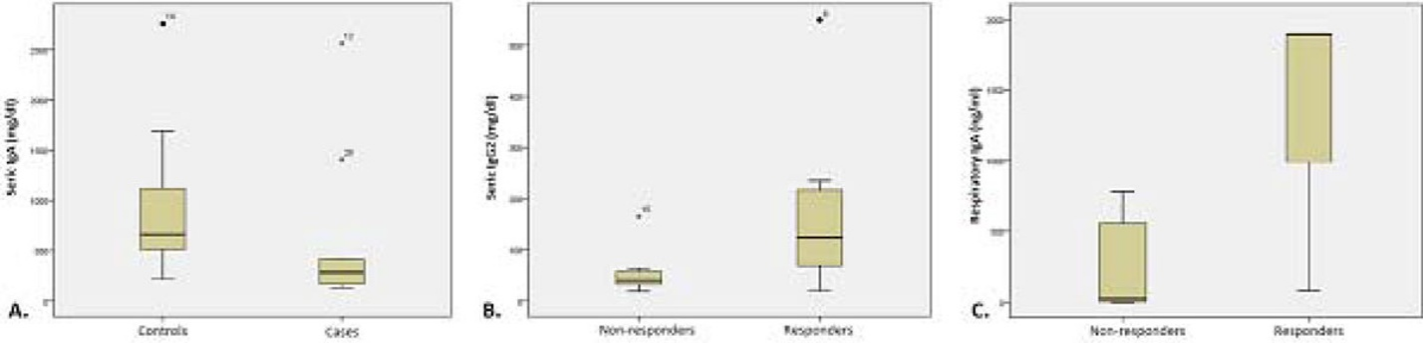
**Seric and respiratory Ig levels**.

